# HIF-1α-Deficiency in Myeloid Cells Leads to a Disturbed Accumulation of Myeloid Derived Suppressor Cells (MDSC) During Pregnancy and to an Increased Abortion Rate in Mice

**DOI:** 10.3389/fimmu.2019.00161

**Published:** 2019-02-05

**Authors:** Natascha Köstlin-Gille, Stefanie Dietz, Julian Schwarz, Bärbel Spring, Jan Pauluschke-Fröhlich, Christian F. Poets, Christian Gille

**Affiliations:** ^1^Department of Neonatology, Tuebingen University Children's Hospital, Tuebingen, Germany; ^2^Department of Obstetrics and Gynecology, Tuebingen, Germany

**Keywords:** HIF, pregnancy, MDSC, abortion, apoptosis

## Abstract

Abortions are the most important reason for unintentional childlessness. During pregnancy, maternal immune cells are in close contact to cells of the semi-allogeneic fetus. Dysregulation of the maternal immune system leading to defective adaptation to pregnancy often plays a role in pathogenesis of abortions. Myeloid-derived suppressor cells (MDSC) are myeloid cells that suppress functions of other immune cells, especially T-cells, thereby negatively affecting diseases such as cancer, sepsis or trauma. They seem, however, also necessary for maintenance of maternal-fetal tolerance. Mechanisms regulating MDSC expansion and function during pregnancy are only incompletely understood. In tumor environment, hypoxia is crucial for MDSC accumulation and activation. Hypoxia is also important for early placenta and embryo development. Effects of hypoxia are mediated through hypoxia-inducible factor 1α (HIF-1α). In the present study we aimed to examine the role of HIF-1α in myeloid cells for MDSC accumulation and MDSC function during pregnancy and for pregnancy outcome. We therefore used a mouse model with targeted deletion of HIF-1α in myeloid cells (myeloid HIF-KO) and analyzed blood, spleens and uteri of pregnant mice at gestational day E 10.5 in comparison to non-pregnant animals and wildtype (WT) animals. Further we analyzed pregnancy success by determining rates of failed implantation and abortion in WT and myeloid HIF-KO animals. We found that myeloid HIF-KO in mice led to an abrogated MDSC accumulation in the pregnant uterus and to impaired suppressive activity of MDSC. While expression of chemokine receptors and integrins on MDSC was not affected by HIF-1α, myeloid HIF-KO led to increased apoptosis rates of MDSC in the uterus. Myeloid-HIF-KO resulted in increased proportions of non-pregnant animals after positive vaginal plug and increased abortion rates, suggesting that activation of HIF-1α dependent pathways in MDSC are important for maintenance of pregnancy.

## Introduction

Abortions are one of the most important pregnancy complication; at least 25%, probably up to 50% of women are affected (1). Recurrent Abortions (RA) are defined as three or more consecutive abortions and affect about 1-3% of couples of childbearing age (2). Besides chromosomal abnormalities, anatomic anomalies and infections, a dysregulation of the immune system seems to play an important role (1, 2). During pregnancy, maternal immune cells and cells of the semi-allogeneic fetus are in close contact. To avoid rejection of the fetus, the maternal immune system has tightly to be regulated (3). Mechanisms inducing maternal-fetal tolerance during pregnancy and those leading to complications like recurrent abortions are incompletely understood.

Myeloid derived suppressor cells (MDSC) are myeloid cells with suppressive activity on other immune cells, especially on T-cells (4). Depending on their phenotype, they can be sub-grouped in two populations – monocytic MDSC (MO-MDSC) and granulocytic MDSC (GR-MDSC). In mice, MDSC can be identified by co-expression of the myeloid lineage differentiation antigen Gr-1 and CD11b. MO-MDSC and GR-MDSC can further be differentiated by the expression of the two epitopes of Gr-1, i.e., Ly6G and Ly6C; MO-MDSC are defined as CD11b^+^/Ly6G^−^/Ly6C^high^ and GR-MDSC as CD11b^+^/Ly6G^+^/Ly6C^low^ (5, 6). Primarily, accumulation of MDSC has been described in cancer patients (7, 8) and tumor bearing mice (5), where they inhibit T-cell response against the tumor leading to disease progression. Later, we and others could show that MDSC and especially GR-MDSC also accumulate during pregnancy in the fetal and maternal organism and that their immune-modulatory properties may be crucial for maternal-fetal tolerance (9–13) and immune adaptation of the newborn (14–17). However, only little is known about mechanisms leading to MDSC-accumulation during pregnancy.

Hypoxia plays an important role in normal placental development as well as in pathogenesis of placental pathologies (18, 19). Hypoxia-inducible factor 1 (HIF-1) is a key regulator of the response to hypoxia, initiating transcription of various genes. HIF-1 is a heterodimer consisting of the subunits HIF-1α and HIF-1β; HIF-1β is constitutively expressed, while expression of HIF-1α is induced under hypoxic conditions (20). During early gestation, HIF-1α is highly expressed in the placenta, which is characterized by low oxygen pressure (21). In the context of malignancies HIF-1α may be critical for MDSC-activation in the hypoxic environment of tumors (22–24). Until now, nothing is known about the role of HIF-1α for MDSC accumulation and activation during pregnancy.

In the present study, we evaluated the impact of HIF-1α on MDSC accumulation and activation during pregnancy in a mouse model of HIF-1α deficiency in myeloid cells. We found that HIF-1α deficiency in myeloid cells (myeloid HIF-KO) (1) led to a diminished accumulation of MDSC during pregnancy, especially in the uterus of pregnant mice, (2) MDSC from myeloid HIF-KO mice had lower suppressive activity than wildtype (WT) MDSC, (3) MDSC from myeloid HIF-KO mice exhibited higher apoptosis rates and (4) myeloid HIF-KO mice had increased abortion rates compared to WT animals.

Taken together, we describe a role of HIF-1α for MDSC accumulation and function during pregnancy and for pregnancy maintenance. Disturbed activation of HIF-1α and resulting alterations in MDSC homeostasis during pregnancy may be a yet unknown mechanism for immunological pregnancy complications.

## Methods

### Mice

HIF-1α^flox^ (B6.129-Hif1a^tm3Rsjo^/J) mice and LysMcre (B6.129P2-*Lyzs*^*tm*1(*cre*)*Ifo*^/J) mice were obtained from The Jackson Laboratory (Bar Harbor, Maine, USA). HIF-1α^flox^ mice and LysMcre mice were crossed to get animals double homozygous for HIF-1α^flox^ and LysMcre, carrying a deletion of HIF-1α in myeloid cells (HIF-1α^flox^/LysMre, myeloid HIF-KO). C57BL/6J (WT) mice were also obtained from The Jackson Laboratory. All animals were maintained under pathogen-free conditions in the research animal facility of Tuebingen University, Tuebingen, Germany. All experimental animal procedures were conducted according to German federal and state regulations.

Syngeneic matings of myeloid HIF-KO and WT mice were set up at 8-12 weeks of age. Gestational ages were determined by visualizing the presence of a vaginal plug (E0.5 = vaginal plug day).

Abortion rates were determined by visual inspection of fetal-placental units and defined as ratio between resorbing units and total implantation sites. Resorbing units were either dark, small and necrotic or pale, small and with no visible fetus inside the amniotic cavity.

### Tissue Collection and Single Cell Preparations

Non-pregnant and pregnant mice at gestational age E10.5 were euthanized by CO_2_ inhalation. Blood (0.5–1 ml) was collected by intracardial puncture immediately after death and placed into EDTA-tubes. Red blood cells were removed from whole blood by Ammonium chloride lysis. Spleens were removed and tissue was pushed through a 100 μm filter (Greiner bio-one, Frickenhausen, Germany) using a syringe plunger. Red blood cells were removed by Ammonium chloride lysis and the resulting cell suspension was then passed again through a 40 μm filter (Greiner bio-one, Frickenhausen, Germany) Uterine horns were removed in toto. The fetuses and the fetal part of placenta were dissected from the uteri and blood vessels were removed. Uteri were then placed into PBS, minced into ~1 mm^3^ pieces and pushed through a 40 μm filter. All cell suspensions were then adjusted to 1–4 × 10^6^ cells/ml in PBS.

### *In-vitro* Generation of MDSC

*in-vitro* generation of MDSC was performed according to previously established protocols (25, 26). For *in-vitro* generation of MDSC non-pregnant WT and myeloid HIF-KO mice were euthanized and femora removed. Bone marrow was collected by rinsing the bones with PBS with a syringe and a 25G needle. Bone marrow cells were then washed, adjusted to 3x10^5^ cells/ml and cultured for 72 h at 37°C in culture medium [Dulbecco's modified eagle medium, DMEM (Thermo Fisher Scientific, Darmstadt, Germany), supplemented with 10% fetal calf serum (FCS, Biochrom, Berlin, Germany) and 1% Penicilline/Streptomycin (Biochrom, Berlin, Germany)] supplemented with 100 ng/ml recombinant murine G-CSF (Peprotech, Hamburg, Germany) and 12.5 ng/ml recombinant murine GM-CSF (Peprotech, Hamburg, Germany). After 72 h of culture non-adherent cells were removed and adherent MDSC detached with trypsin (Biochrom GmbH, Berlin, Germany). >90% of cells were Gr-1^+^/CD11b^+^ as determined by flow cytometry, thereby exhibiting surface characteristics of MDSC.

### Cell Isolation and Flow Cytometry

For isolation of CD4^+^ from splenocytes, cells were labeled with T-cell Biotin-Antibody Cocktail followed by two sequential Anti-Biotin magnetic bead separation steps (Miltenyi Biotec, Bergisch-Gladbach, Germany) according to the manufacturer's instructions. Purity of CD4^+^ T-cells after separation was >90%, as assessed by flow cytometry.

For extracellular staining, freshly isolated cells were washed in washing buffer [PBS with 0.1% bovine serum albumin (BSA)] and FcRs were blocked with purified anti-CD16/32 (clone 2.4G2) for 10 min. Then, fluorescent-conjugated extracellular antibodies were added. Antibodies were purchased from BD biosciences [CD3 (145-2C11), CD4 (RM4-5), CD8a (53-6.7), CD11b (M1/79), CD19 (1D3), CD45 (30-F11), NK1.1 (PK136), Gr-1 (RB6-8C5), Ly-6C (AL-21), Ly-6G (1A8), CXCR5 (2G8), annexin V] and R&D systems [CXCR1 (FAB8628P), CXCR2 (FAB2164C), CXCR4 (FAB21651C), CXC3CR1 (FAB5825P), IL4-Rα (FAB530P), Integrin-α4 (FAB2450P) Integrin-β2 (FAB2618P), L-Selectin (FAB5761P)]. For immune cell quantification, cells were pre-gated to CD45. Among CD45^+^ cells, cell types were identified as follows: T-cells CD3^+^, T-Helper cells CD3^+^/CD4+, cytotoxic T-cells CD3^+^/CD8^+^ B-cells CD3^−^/CD19^+^, NK-cells CD3^−^/NK1.1^+^, MDSC CD11b^+^/Gr-1^+^, MO-MDSC CD11b^+^/Ly6C^+^/Ly6G^−^, GR-MDSC CD11b^+^/Ly6C^low^/Ly6G^+^, monocytes CD11b^+^/Gr-1^−^.

Data acquisition was performed with a FACScalibur flow cytometer (BD Bioscience) and analyzed via CellQuest (BD Biosciences).

### T-Cell Suppression Assay

Freshly isolated CD4^+^ splenocytes were stained with carboxyfluorescein-succinimidyl ester (CFSE, Invitrogen, Heidelberg, Germany) according to the manufacturer's instructions. Cells were suspended in RPMI 1640 media containing 1% penicillin/streptomycin and 10% FCS. CFSE-labeled CD4^+^ T-cells (2 × 10^5^) suspended in 100 μl media were stimulated with 2 × 10^5^ mouse T-Activator CD3/CD28 Dynabeads (Thermo Fisher Scientific, Dreieich, Germany) and 50 ng recombinant murine Interleukin-2 (rmIL-2, R&D Systems, Wiesbaden-Nordenstadt, Germany). *In-vitro* generated MDSC also suspended in RPMI 1640 containing 1% penicillin/streptomycin and 10% FCS were added in different ratios (1:1, 1:2 and 1:4). After 5 days of culture, CD4^+^ T-cell proliferation was determined by CFSE dye dilution by flow cytometry. Proliferation index, defined as the ratio of CD4^+^ T-cell proliferation after addition of MDSC and CD4^+^ T-cell proliferation without MDSC, was determined. CD4^+^ T-cell proliferation without MDSC was set to a fixed value of 1.

### Statistical Analysis

Statistical analysis was done using GraphPad Prism 5.0 (GraphPad Software, La Jolla, CA). Data were analyzed for Gaussian distribution using D'Agostino and Pearson omnibus normality test. Immune cell quantification experiments were analyzed using the Kruskal Wallis test and Dunn's multiple comparison test. T-cell suppression was analyzed using the Wilcoxon matched pairs signed rank test. Expression of surface receptors and apoptosis rates were analyzed using the Mann-Whitney test. Data analyzing pregnancy success (rate of pregnant animals after positive vaginal plug and rate of animals with at least one abortion) were analyzed by Fishers exact test. Abortion rate was analyzed by Mann-Whitney test. Normally distributed data were analyzed using paired *t*-test, not normally distributed data were evaluated using the Wilcoxon matched pairs signed rank test. A *p*-value < 0.05 was considered as statistically significant.

## Results

### HIF-1α Deficiency in Myeloid Cells Leads to a Diminished Accumulation of MDSC in the Pregnant Uterus

**Figure 1:**
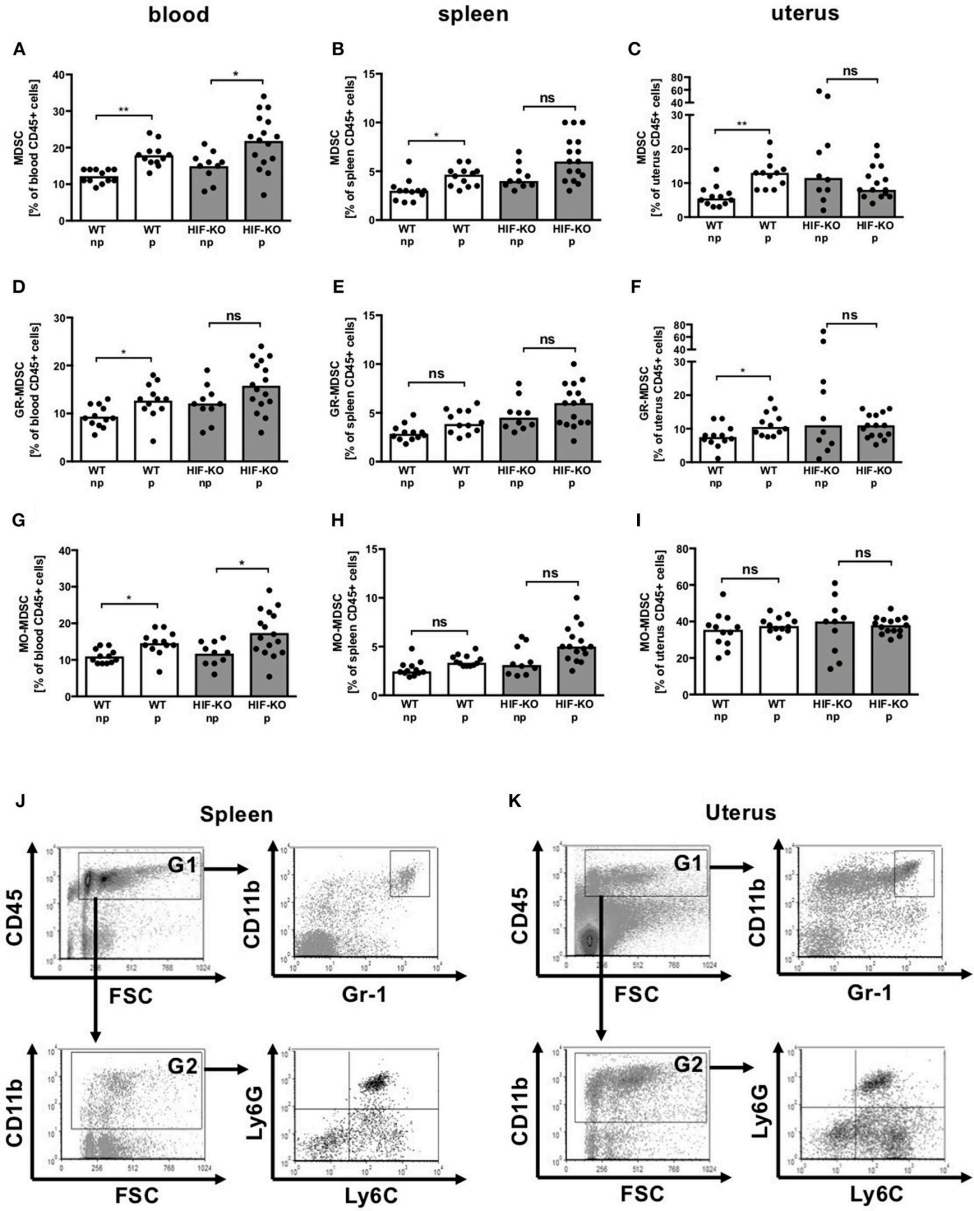
Quantification of MDSC in blood, spleens and uteri of wildtype and myeloid HIF-KO mice. Non-pregnant (np) and E10.5 pregnant (p) wildtype (WT) and myeloid HIF-KO (HIF-KO) mice were euthanized and blood, spleens and uteri were collected. Tissues were homogenized and filtered to obtain single cell suspensions. Cells were then analyzed by flow cytometry. **(A–I)** Scatter diagrams with bars showing percentages of total MDSC **(A–C)** GR-MDSC **(D–F)**, and MO-MDSC **(G-I)** from total CD45^+^ leucocytes in peripheral blood **(A,D,G)**, spleens **(B,E,H)** and uteri **(C,E,I)**. Each symbol represents an individual sample and the median is indicated. *n* = 10–16, *p < 0.05; **p < 0.01; ns not significant; Kruskal-Wallis test and Dunn's multiple comparison test. **(J,K)** Representative density plots showing the gating strategy for total MDSC (CD11b^+^/Gr-1^+^), GR-MDSC (CD11b^+^/Ly6C^low^/Ly6G^+^) and MO-MDSC (CD11b^+^/Ly6C^high^/Ly6G^−^) in spleens **(J)** and uteri **(K)**.

HIF-1α has been shown to be essential for MDSC function in the tumor environment (22). Therefore, we first asked whether HIF-1α plays a role for MDSC accumulation also during pregnancy. We analyzed expression of MDSC in blood, spleens and uteri of non-pregnant and pregnant WT mice at embryonic day 10.5 (E10.5) in comparison to myeloid HIF-KO mice. Pregnant WT mice had significantly higher proportions of CD11b^+^/Gr-1^+^ MDSC in blood, spleen and uteri than non-pregnant WT mice (Figures 1A–C). In myeloid HIF-KO mice, MDSC counts in non-pregnant animals tended to be higher in blood, spleen and uterus compared to WT mice. Pregnancy induced further MDSC accumulation only in blood, not in spleens or uteri. In the latter, we even found a tendency toward lower MDSC numbers in pregnant than in non-pregnant myeloid HIF-KO mice (Figures 1A–I). MDSC-accumulation in WT animals was mainly attributed to the CD11b^+^/Ly6G^+^/Ly6C^low^ GR-MDSC subpopulation (Figures 1D–F), not the CD11b^+^/Ly6G^−^/Ly6C^high^ MO-MDSC subpopulation (Figures 1G–I). Gating strategy for MDSC, GR-MDSC and MO-MDSC in spleens and uteri are depicted in Figures 1J–K.

Regarding other leucocyte subpopulations, we found a decrease in percentages of B-cells in blood and NK-cells in spleens of both WT-mice and myeloid HIF-KO-mice during pregnancy (Supplementary Figures 1D,H). Percentages of T-cells decreased in uteri of myeloid HIF-KO mice during pregnancy, but remained unchanged in WT-mice (Supplementary Figure 1C). Percentages of monocytes increased in blood and spleens of pregnant myeloid HIF-KO but not WT-mice (Supplementary Figures 1J,K), while they decreased in uteri of pregnant WT-mice and remained unchanged in uteri of myeloid HIF-KO mice (Supplementary Figure 1L).

### HIF-1α Deficiency Leads to Diminished Suppressive Activity of MDSC

**Figure 2:**
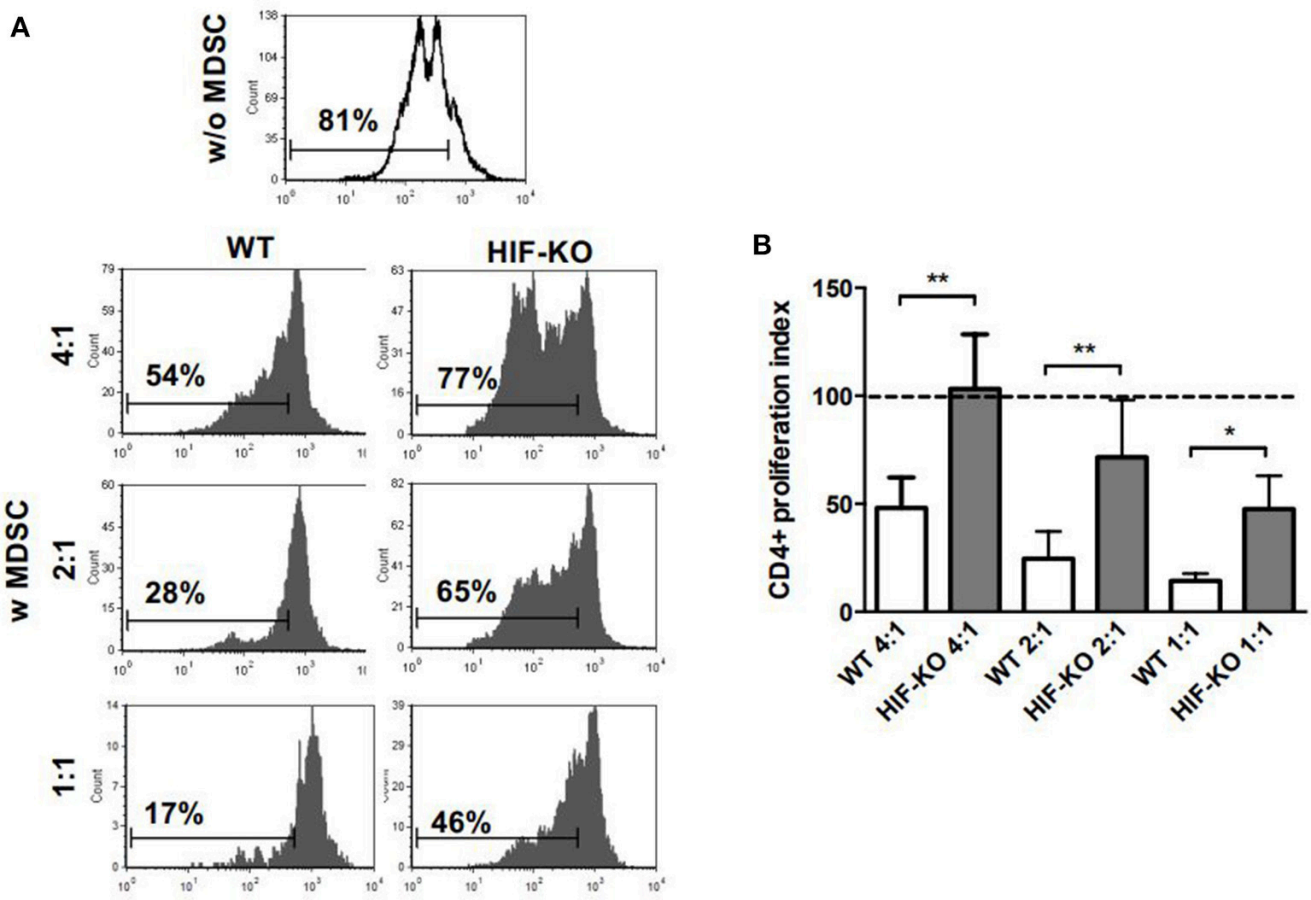
Inhibition of CD4^+^ T-cell proliferation by MDSC generated from wildtype and myeloid HIF-KO mice. Non-pregnant wildtype (WT) mice and myeloid HIF-KO mice (HIF-KO) were euthanized and bone marrow cells were collected. Cells were cultured for 4 days with G-CSF and GM-CSF. After 4 days non-adherent cells were removed and adherent MDSC were detached with Trypsin/EDTA. MDSC were then added to CD4^+^ T-cells, freshly isolated from spleens of non-pregnant wildtype mice by MACS, stained with CFSE and stimulated with anti-CD3/CD28 microbeads. After 4 days, proliferation of CD4^+^ T-cells was assessed by CFSE dye dilution. Proliferation index was determined as ratio of T-cell proliferation with and without addition of MDSC. **(A)** Representative histogram plots showing proliferation of CD4^+^ T-cells without (white histogram, w/o MDSC) addition of MDSC and with (gray histograms, w MDSC) addition of MDSC generated from WT mice (left side) and from HIF-KO mice (right side) in T-cell:MDSC ratios of 4:1, 2:1 and 1:1. **(B)** Inhibitory effect of MDSC from WT mice (white bars) and HIF-KO mice (gray bars) on proliferation of CD4^+^ T-cells. Dashed line shows proliferation of target CD4^+^ T-cells without addition of MDSC. Inhibition of T-cell proliferation by MDSC was measured at the indicated ratios by CFSE dye dilution. Bars show mean and standard deviation of 5 samples pooled from 5 independent experiments. *p < 0.05; **p < 0.01 compared with target cells alone; Wilcoxon matched-pairs signed-rank test.

Next, we asked whether HIF-1α deficiency also has effects on suppressive activity of MDSC. To test this hypothesis, we generated MDSC from bone marrow (BM) of WT mice and myeloid HIF-KO mice and tested their suppressive activity on CD4^+^ T-cell proliferation. MDSC generated from BM of WT mice efficiently suppressed CD4^+^ T-cell proliferation in a concentration dependent manner to 48.0 ± 14.2 % (1:4), 24.6% ± 12.5% (1:2) and 14.3% ± 3.4% (1:1). In contrast, MDSC generated from BM of myeloid HIF-KO mice displayed substantially lower suppressive activity on CD4^+^ T-cell (103.2% ± 25.2% (4:1) *p* < 0.01 vs. WT mice, 71.6% ± 26.5% (2:1) *p* < 0.01 vs. WT mice and 47.5% ± 15.4% (1:1) *p* < 0.05 vs. WT mice, *n* = 5, Figures 2A,B).

### Diminished Accumulation of MDSC in the Pregnant Uterus Is Not Due to an Altered Expression of Chemokine Receptors or Integrins

**Figure 3:**
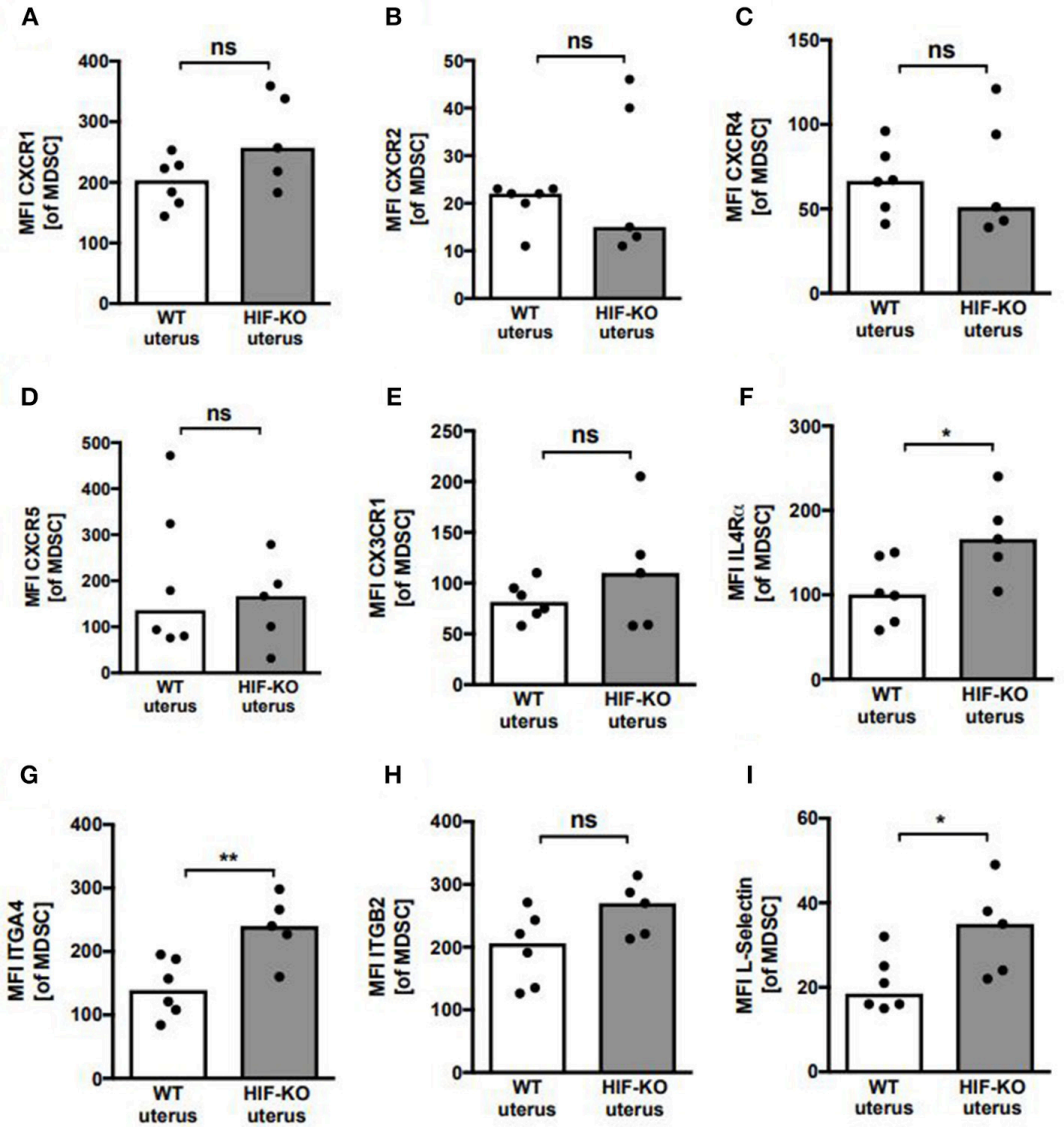
Expression of chemokine receptors and integrins on uterine MDSC from wildtype and myeloid HIF-KO mice. **(A–I)** E10.5 pregnant wildtype (WT) and myeloid HIF-KO (HIF-KO) mice were euthanized and uteri were collected. Tissues were homogenized and filtered to obtain single cell suspensions. Cells were then analyzed by flow cytometry. Scatter diagrams with bars showing MFI for indicated chemokine receptor and integrin expression on CD11b^+^/Gr-1^+^ MDSC from wildtype (white bars) and myeloid HIF-KO (HIF-KO) mice. Each symbol represents an individual sample and the median is indicated. *n* = 5–6, *p < 0.05; **p < 0.01; ns, not significant; Mann-Whitney test.

As the most impressive difference between WT mice and myeloid HIF-KO mice was a diminished influx of MDSC into the pregnant uterus in myeloid HIF-KO mice, we asked whether deficiency for HIF-1α might lead to a diminished expression of chemokine receptors or integrins on MDSC leading to defects in their migratory capacity. Therefore, we analyzed expression of chemokine receptors CXCR1 (CD181), CXCR2 (CD182), CXCR4 (CD84), CXCR5 (CD185), CX3CR1 and IL4Rα (CD124), as well as ITGA4 (CD49d), ITGB2 (CD18) and L-selectin (CD62L) in MDSC from spleens and uteri of WT and myeloid HIF-KO mice. We found no differences in the expression of CXCR1, CXCR2, CXCR4, CXCR5, and CX3CR1 between WT and myeloid HIF-KO mice, neither in splenic MDSC nor in uterine MDSC. Interestingly, we even found higher expression of IL4Rα, ITGA4, ITGB2, and L-Selectin on myeloid HIF-KO MDSC (Supplementary Figure 2 and Figure 3), not explaining the diminished accumulation in pregnant uteri.

### HIF-1α Deficiency in Myeloid Cells Leads to Increased Apoptosis Rates of MDSC

**Figure 4:**
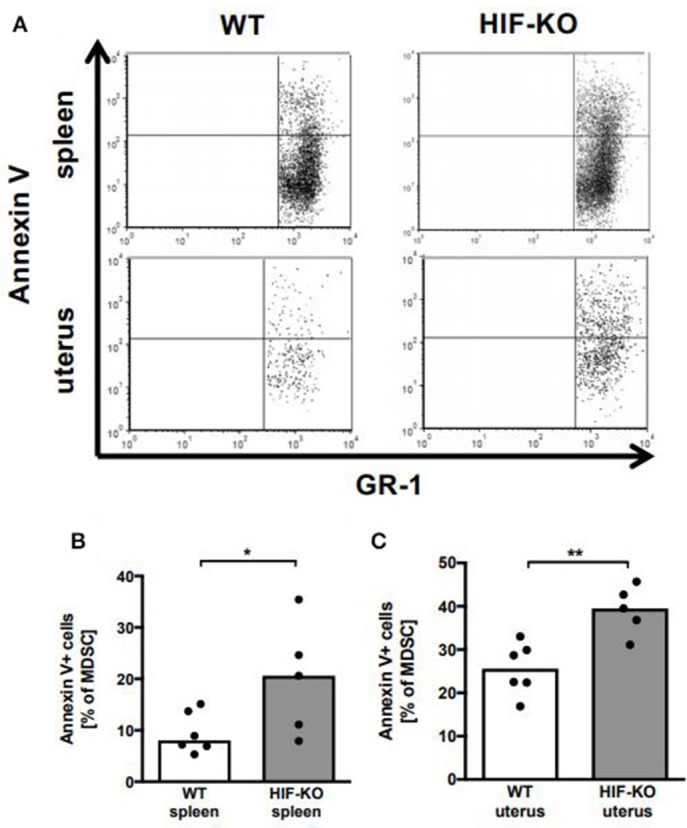
Apoptosis of MDSC from spleens and uteri from wildtype and myeloid HIF-KO mice. E10.5 pregnant wildtype (WT) and myeloid HIF-KO (HIF-KO) mice were euthanized and spleens and uteri were collected. Tissues were homogenized and filtered to obtain single cell suspensions. Cells were then analyzed for apoptosis annexin V staining and flow cytometry. **(A)** Representative density plots from spleens (upper plots) and uteri (lower plots) of Wildtype (WT) and myeloid HIF-KO (HIF-KO) mice for GR-1 vs. annexin V. **(B,C)** Scatter diagrams with bars showing percentages of annexin V^+^ MDSC from total MDSC in spleens **(B)** and uteri **(C)** from wildtype (white bars) and myeloid HIF-KO (HIF-KO) mice. Each symbol represents an individual sample and the median is indicated. *n* = 5–6, *p < 0.05; **p < 0.01; Mann-Whitney test.

To further figure out the mechanism(s) underlying the diminished accumulation of MDSC in uteri of pregnant myeloid HIF-KO mice, we analyzed apoptosis rates of MDSC in spleens and uteri of pregnant WT and myeloid HIF-KO mice by annexin V staining. We found that apoptosis rates of MDSC from spleens and uteri of myeloid HIF-KO mice were about twice as high as those of WT-MDSC (median 20.6 vs. 8.1% for spleens and 39.5 vs. 25.6% for uteri, *n* = 5–6, *p* < 0.05, Figures 4A–C).

### HIF-1α Deficiency in Myeloid Cells Leads to an Increased Abortion Rate in Mice

**Figure 5:**
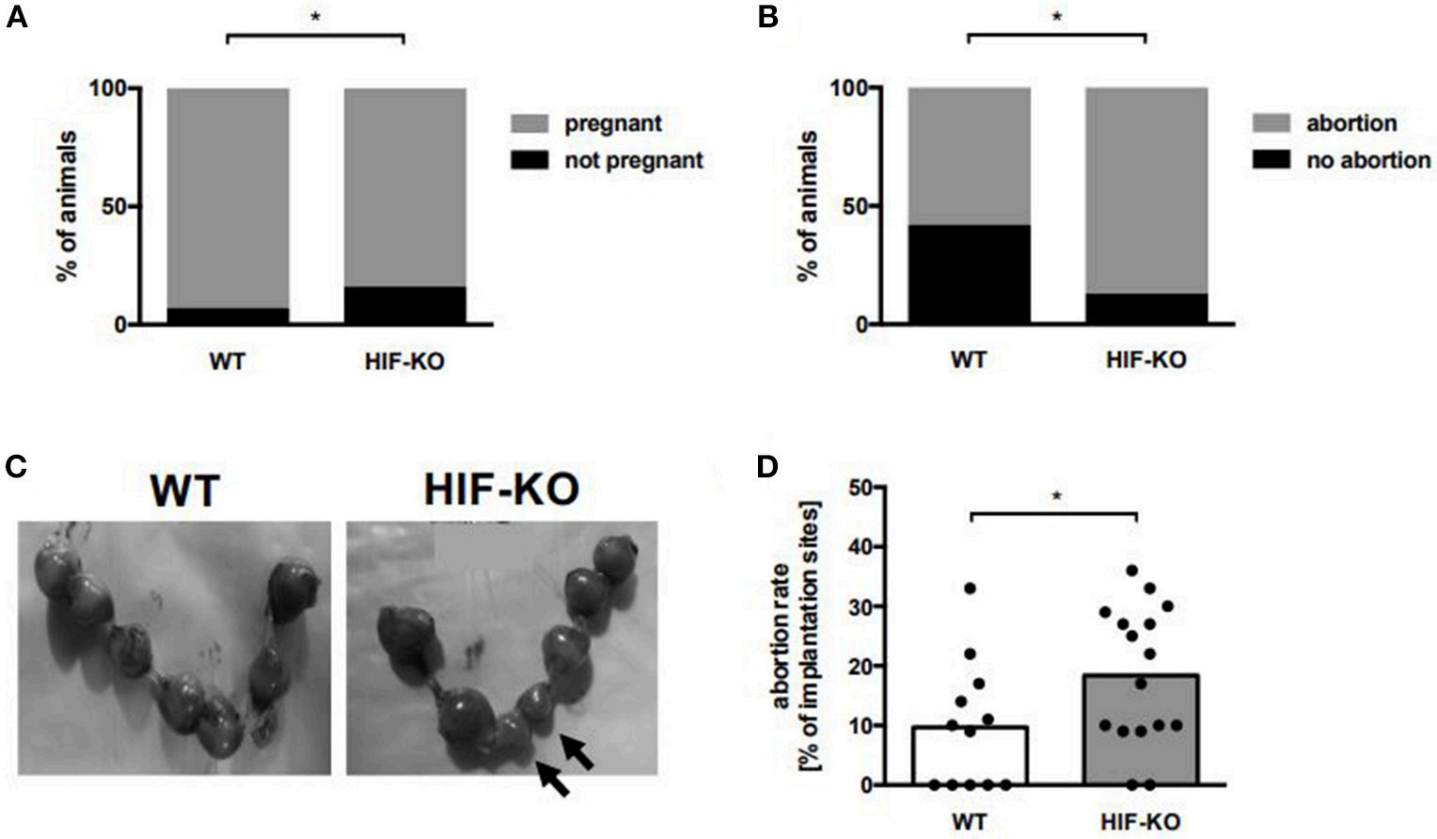
Pregnancy success in wildtype and myeloid HIF-KO mice. Wildtype and myeloid HIF-KO mice were term-bred and the day when a vaginal plug was detected was defined as day E0.5. At day E10.5 mice were euthanized and the abdominal cavity was opened. Uteri containing foeto-placental units were removed and inspected. Total implantation sides and resorbing units were counted. **(A)** Bar graph showing the percentages of non-pregnant (black part) and pregnant (gray part) animals among wildtype (WT) and myeloid HIF-KO (HIF-KO) mice after positive evaluation for vaginal plug. *n* = 13–20, *p < 0.05, Fishers exact test. **(B)** Bar graph shows the percentages of individuals without (black part) and with (gray part) at least one abortion among wildtype (WT) and myeloid HIF-KO (HIF-KO) mice. *n* = 12–16, *p < 0.05, Fishers exact test. **(C)** Representative pictures of uteri from a pregnant wildtype (WT) and a pregnant myeloid HIF-KO mouse (HIF-KO). Arrows indicate resorbing units. **(D)** Scatter diagram with bars showing abortion rates of pregnant wildtype (white bar) and pregnant myeloid HIF-KO mice (gray bar). Abortion rates were defined as ratio between resorbing units and total implantation sites. Each symbol represents an individual animal and the median is indicated. *n* = 12–16, *p < 0.05; Mann-Whitney test.

To evaluate the clinical significance of myeloid HIF-KO during pregnancy, we analyzed pregnancy outcomes in myeloid HIF-KO mice and WT mice at E10.5. Placentae were prepared and numbers of resorbing units quantified. Resorbing units were either dark, small and necrotic, pale or small and with no visible fetus inside the amniotic cavity. We found that the rate of non-pregnant animals after positive vaginal plug was significantly higher in myeloid HIF-KO animals (16 vs. 7%, *n* = 13–20, *p* < 0.05, Fishers exact test, Figure 5A). Furthermore, we found a higher rate of animals with at least one abortion in myeloid HIF-KO animals than in WT mice (87 vs. 58%, *n* = 12–16, *p* < 0.0001, Fishers exact test, Figure 5B). At day E10.5, we found no differences in the number of implantation sites between myeloid HIF-KO mice and WT mice (Supplementary Figure 3), however, myeloid HIF-KO mice had significant higher abortion rates than WT mice (median 19.5% vs. 9.5%, *n* = 12–16, *p* < 0.05, Figures 5C,D).

### Discussion

The role of MDSC in maintaining pregnancy is increasingly recognized. Molecular mechanisms leading to MDSC-accumulation during pregnancy, however, are still incompletely understood. In the present study, we investigated the role of the hypoxia-regulated transcription factor HIF-1α for MDSC-accumulation during pregnancy and for pregnancy outcome.

First, we found that knockout of HIF-1α in myeloid cells led to a diminished accumulation of MDSC in the pregnant uterus, illustrating a relationship between myeloid HIF-1α expression and MDSC accumulation during pregnancy. One other study recently described a HIF-1α dependent accumulation of MDSC under tumor conditions; in a mouse model of hepatocellular carcinoma, HIF-1α activation in tumor cells led to an overexpression of the ectonucleotidase CD39 mediating a differentiation arrest of MDSC thereby leading to their accumulation (27). Other studies investigating the interplay between HIF-1α-activation and MDSC focused on MDSC function and not on MDSC accumulation (22–24). Interestingly, besides the uterine milieu, where hypoxia is known to play a role during different stages of pregnancy (18, 19), we also found a diminished MDSC-accumulation in spleens of myeloid HIF-KO mice. This suggests that, in addition to hypoxia, other factors may activate HIF-1α during pregnancy. Besides inflammatory pathways such as NF-κB that has been described to activate HIF-1α (28), one study reported an activation of HIF-1α by the sex hormone estrogen (29). Furthermore it has been shown that *in vitro* estrogen activates MDSC during pregnancy (30). The impact of estrogen on HIF-1α activation in MDSC and their functional activation during pregnancy is content of ongoing studies.

Interestingly, MDSC-accumulation in the pregnant uterus of wildtype mice was accompanied by a reduction of uterine monocytes. This reduction was not observed in uteri of myeloid HIF-KO mice. Since MDSC can differentiate to mature myeloid cells and hypoxia has been described to prevent MDSC differentiation via HIF-1α (27), the decreased monocyte numbers in pregnant uteri of WT mice might be a result of a HIF-1α-driven maturational arrest.

Second, we found that MDSC generated from myeloid HIF-KO mice had substantially reduced suppressive activity compared to MDSC generated from WT mice. This is in line with other studies describing an increased suppressive activity of MDSC under hypoxic tumor conditions mediated by HIF-1α (22). We now show that lack of HIF-1α leads to impaired suppressive activity of MDSC also in normoxia. The underlying mechanism for reduced suppressive activity of HIF-KO MDSC remains unclear. One of the main effector mechanisms used by MDSC to suppress T-cells is the production of iNOS (6). It has been shown that the Th1-cytokine IFN-γ activates HIF-1α leading to increased iNOS expression (31) and that myeloid cells acquire suppressive activity under hypoxic tumor conditions that can be abrogated by inhibiton of iNOS (23) so that it could be speculated that decreased generation of NO in HIF-KO MDSC leads to their decreased suppressive activity. Another mechanism linking HIF-1α activation and MDSC-function is the expression of the immune checkpoint molecule PD-L1. Noman et al. showed that expression of PD-L1 on myeloid cells can be stimulated by Hypoxia via HIF-1α and that blockade of PD-L1 decreases MDSC-mediated T-cell suppression by down-regulating MDSC IL-6 and IL-10 (24). Our group showed that expression of PD-L1 on human MDSC can be stimulated by E.coli (32). Thus, stimulation of PD-L1 expression via HIF-1α could also be a mechanism for MDSC activation during pregnancy.

To figure out mechanisms leading to the disturbed accumulation of MDSC in the pregnant uterus of myeloid HIF-KO mice we analyzed expression of chemokine receptors and integrins on MDSC. Although upregulation via HIF-1α signaling pathways has been described for many surface receptors including CXCR1 and CXCR2 (33), CXCR4 (34), and IL-4Rα (35), we found no differences in their expression on MDSC from WT and myeloid HIF-KO mice. ITGB2 and ITGA4 were found to be upregulated in HIF-KO MDSC confirming other studies that showed negative regulation of ITGA4 by HIF-1α (36) but not explaining the reduced MDSC-accumulation in the uterus. Taken together, our results suggest that HIF-1α knockout in myeloid cells does not alter MDSC migration to the uterus.

We found increased apoptosis rates of MDSC isolated from spleens and uteri of myeloid HIF-KO mice compared to WT mice. This is in line with most previous studies indicating that HIF-1α promotes survival in cancer and endothelial cells (37–41). In contrast, there are also studies describing a proapoptotic role for HIF-1α (42, 43). During pregnancy, data on the role of HIF-1α for apoptosis are conflicting (44, 45). However, previous studies focused on placental cells and not immune cells in the uterus. Regarding our results, the increased apoptosis rates in HIF-KO MDSC may explain their reduced accumulation in the pregnant uterus. Correspondingly, in peripheral organs, myeloid HIF-KO MDSC still expanded, albeit to a lesser extent than WT-MDSC.

Last, we show that myeloid HIF-KO mice had higher rates of non-pregnant animals after positive vaginal plug and higher abortion rates than WT-mice pointing to the potential clinical relevance of reduced MDSC accumulation in the uterus. General loss of HIF-1α causes severe failure in placental formation, resulting in embryo lethality by E10.5. In these animals, placental defects are mainly caused by disturbed development of vascularization and disruption of trophoblast differentiation (46–48). In the embryo, HIF-1α is required for heart development, chondrogenesis and bone formation (49–51). Clinical studies showed upregulation of HIF-1α in trophoblasts of patients with missed abortions (52, 53), and increased HIF-1α activity in placental tissues has been associated with preeclampsia (54–56). However, reports on HIF-1α-regulation in myeloid cells during pregnancy are lacking. The role of hypoxia and HIF-1α in myeloid cells during other inflammatory processes is inconclusive. In a mouse model of LPS-induced Sepsis, HIF-1α induced a proinflammatory phenotype in monocytes and deletion of HIF-1α led to improved survival (57). In dendritic cells, hypoxia and HIFs are described to mediate both, proinflammatory and anti-inflammatory properties (58, 59). Under tumor conditions however, hypoxia and HIF-1α seem to drive the development and function of immunosuppressive myeloid cells like TAMs and MDSCs (23, 60, 61). Correspondingly, a recent study showed that overexpression of HIF-1α in myeloid cells leads to diminished transplant rejection and induction of a regulatory phenotype in myeloid cells in a model of heart transplantation (62). To our knowledge, our study is the first describing an impact of myeloid HIF-1α-expression on pregnancy outcome. Several previous studies in humans (11, 63, 64) and mice (10, 64–66) have shown that reduced MDSC accumulation is associated with abortions. Adoptive transfer experiments with abortion-prone mice furthermore showed that abortion rates were reduced by MDSC-transfer (65, 66). Absence of MDSC also led to failed implantation (13). These results together with our finding of a reduced MDSC-accumulation in myeloid HIF-KO mice make it tempting to speculate that diminished MDSC accumulation is responsible for the disturbed course of pregnancy in these animals.

In conclusion, we show here that HIF-1α expression in myeloid cells is required for a successful pregnancy and that loss of HIF-1α in myeloid cells leads to diminished accumulation, increased apoptosis and impaired MDSC function in the pregnant uterus. These results not only enlarge our knowledge about regulation of MDSC-accumulation and function during pregnancy, but may also help to better understand MDSC biology in other hypoxic conditions such as in solide tumors and inflamed tissues. Targeting HIF-1α may be a promising strategy to modulate MDSC-function.

## Ethics Statement

This study was carried out in accordance with the recommendations of the Einrichtung für Tierschutz, Tierärztlichen Dienst und Versuchtierkunde Tübingen. The protocol was approved by the Einrichtung für Tierschutz, Tierärztlichen Dienst und Versuchtierkunde Tübingen and the Regierungspräsidium Tübingen.

## Author Contributions

NK and CG conceptualized and designed the study. NK, SD, JS, and BS performed experiments. NK, SD, and CG analyzed data. NK drafted the initial manuscript. NK, JP-F, CP, and CG reviewed and revised the manuscript. All authors approved the final manuscript as submitted and agreed to be accountable for all aspects of the work.

### Conflict of Interest Statement

The authors declare that the research was conducted in the absence of any commercial or financial relationships that could be construed as a potential conflict of interest.

## Abbreviations

BM: bone marrow
GR-MDSC: granulocytic MDSC
HIF-1α: hypoxia inducible factor 1 α
HIF-KO: HIF-1α knockout
MDSC: myeloid derived suppressor cells
MO-MDSC: monocytic MDSC
NK: natural killer
WT: wildtype.
