# Supplementary material for: HIF-1α-Deficiency in Myeloid Cells Leads to a Disturbed Accumulation of Myeloid Derived Suppressor Cells (MDSC) During Pregnancy and to an Increased Abortion Rate in Mice
Source: Front Immunol. 2019 Feb 5;10:161. doi: 10.3389/fimmu.2019.00161 (PMC6370686; doi:10.3389/fimmu.2019.00161)
Supplement: Supplementary file 1 [file Presentation_1.PPTX]

## Slide 1
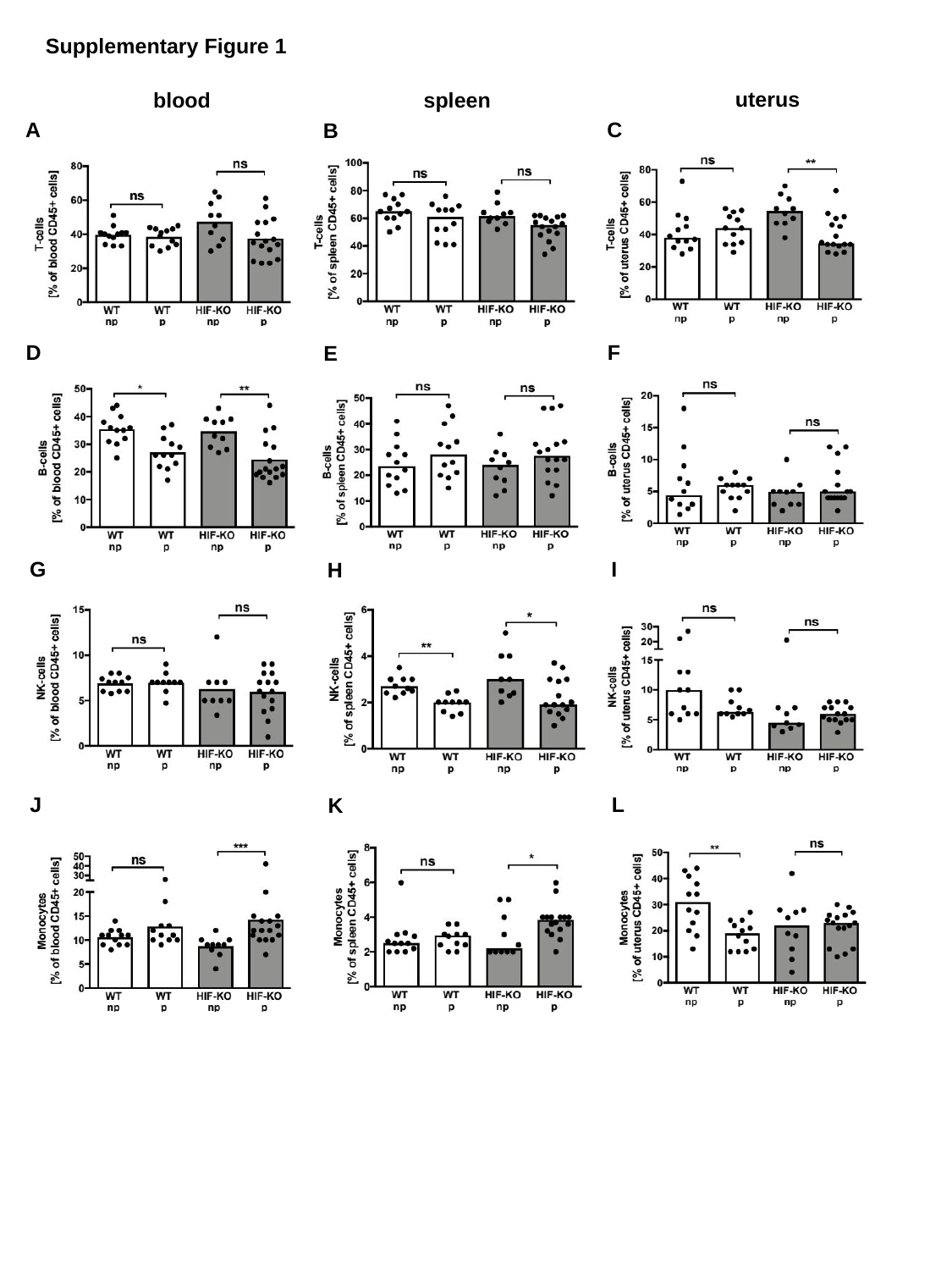

Supplementary Figure 1
uterus
spleen
blood
A
C
B
D
F
E
G
I
H
J
L
K

## Slide 2
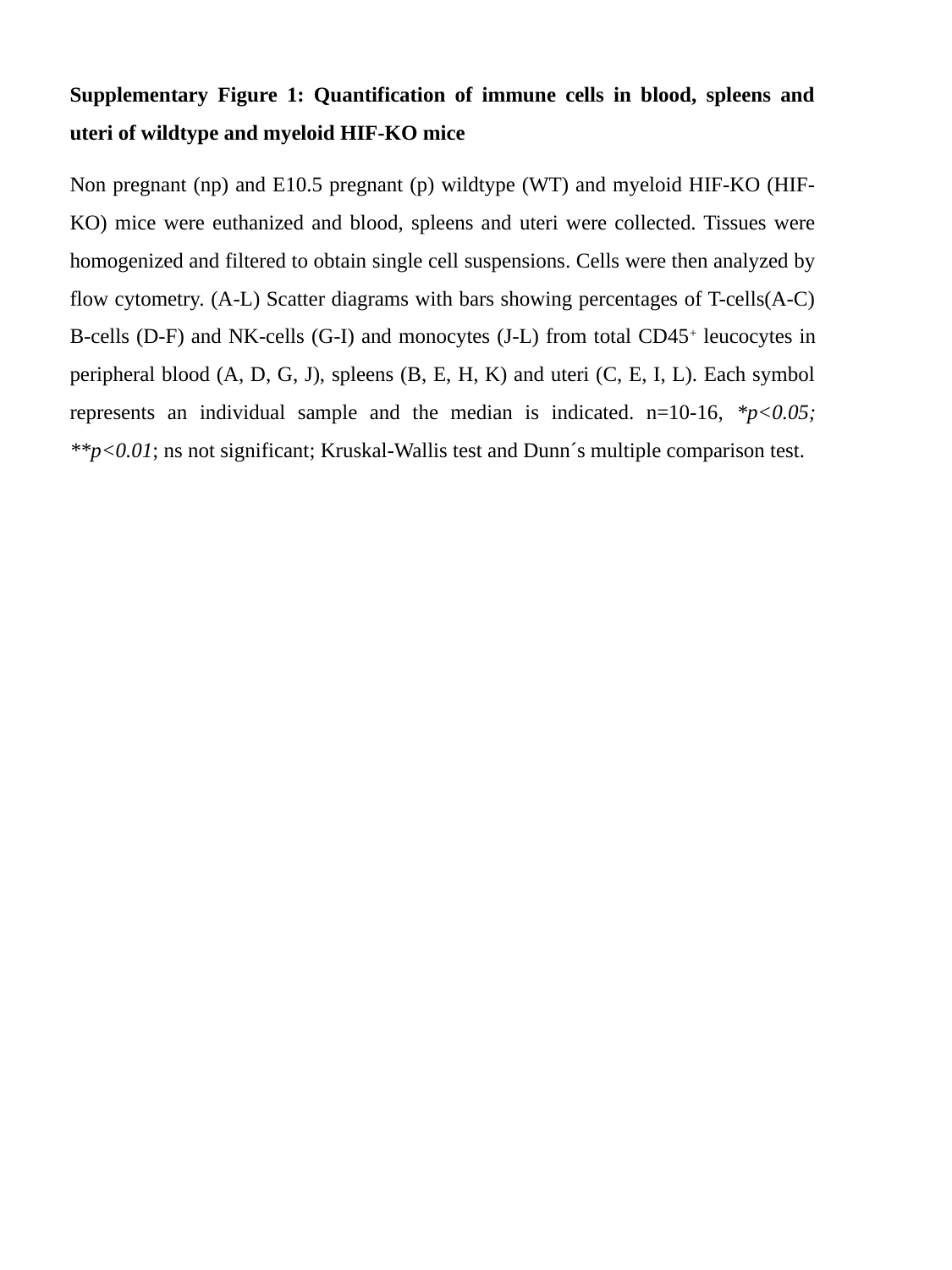

Supplementary Figure 1: Quantification of immune cells in blood, spleens and uteri of wildtype and myeloid HIF-KO mice
Non pregnant (np) and E10.5 pregnant (p) wildtype (WT) and myeloid HIF-KO (HIF-KO) mice were euthanized and blood, spleens and uteri were collected. Tissues were homogenized and filtered to obtain single cell suspensions. Cells were then analyzed by flow cytometry. (A-L) Scatter diagrams with bars showing percentages of T-cells(A-C) B-cells (D-F) and NK-cells (G-I) and monocytes (J-L) from total CD45+ leucocytes in peripheral blood (A, D, G, J), spleens (B, E, H, K) and uteri (C, E, I, L). Each symbol represents an individual sample and the median is indicated. n=10-16, *p<0.05; **p<0.01; ns not significant; Kruskal-Wallis test and Dunn´s multiple comparison test.

## Slide 3
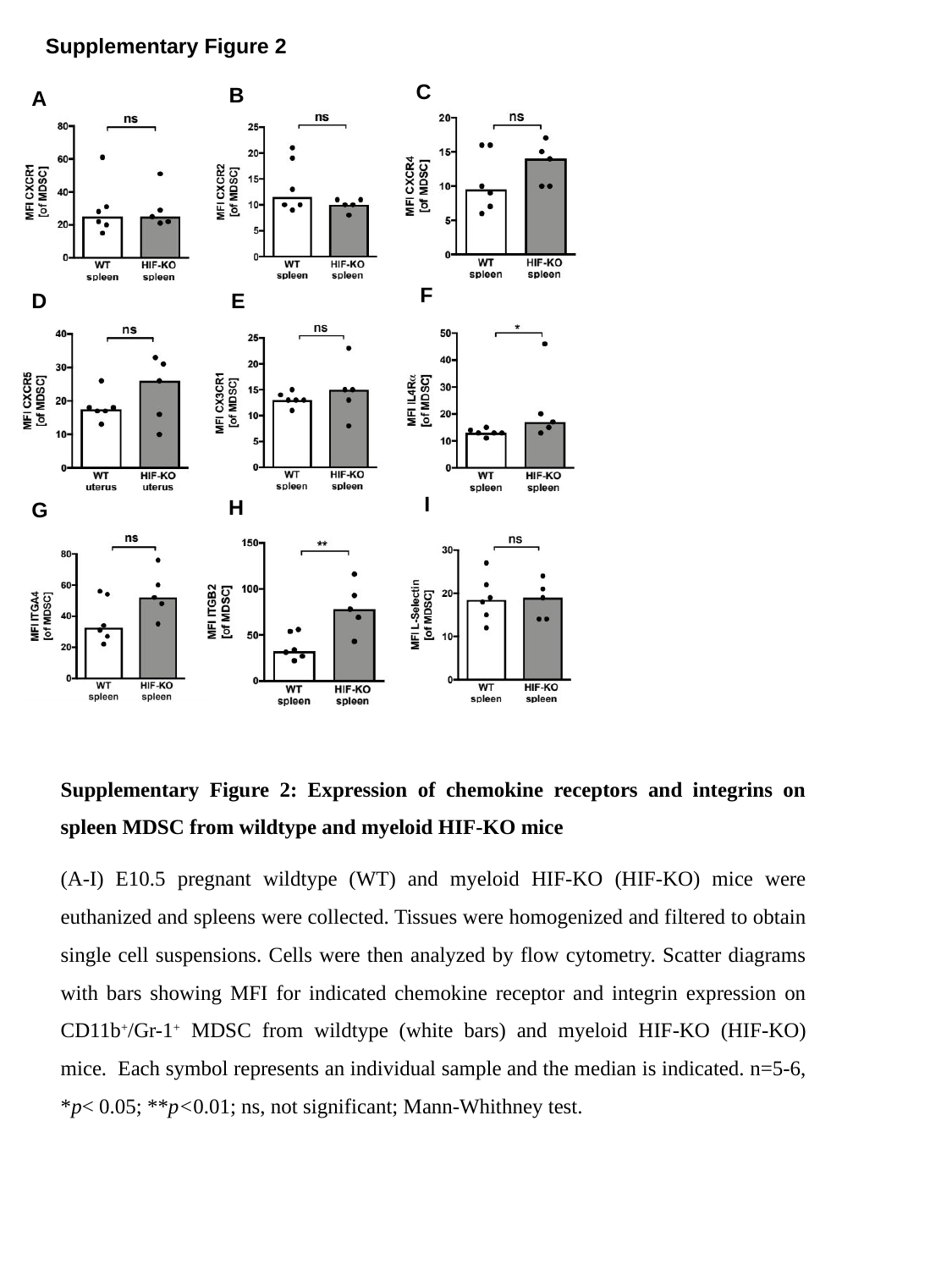

Supplementary Figure 2
C
B
A
F
D
E
I
H
G
Supplementary Figure 2: Expression of chemokine receptors and integrins on spleen MDSC from wildtype and myeloid HIF-KO mice
(A-I) E10.5 pregnant wildtype (WT) and myeloid HIF-KO (HIF-KO) mice were euthanized and spleens were collected. Tissues were homogenized and filtered to obtain single cell suspensions. Cells were then analyzed by flow cytometry. Scatter diagrams with bars showing MFI for indicated chemokine receptor and integrin expression on CD11b+/Gr-1+ MDSC from wildtype (white bars) and myeloid HIF-KO (HIF-KO) mice. Each symbol represents an individual sample and the median is indicated. n=5-6, *p< 0.05; **p<0.01; ns, not significant; Mann-Whithney test.

## Slide 4
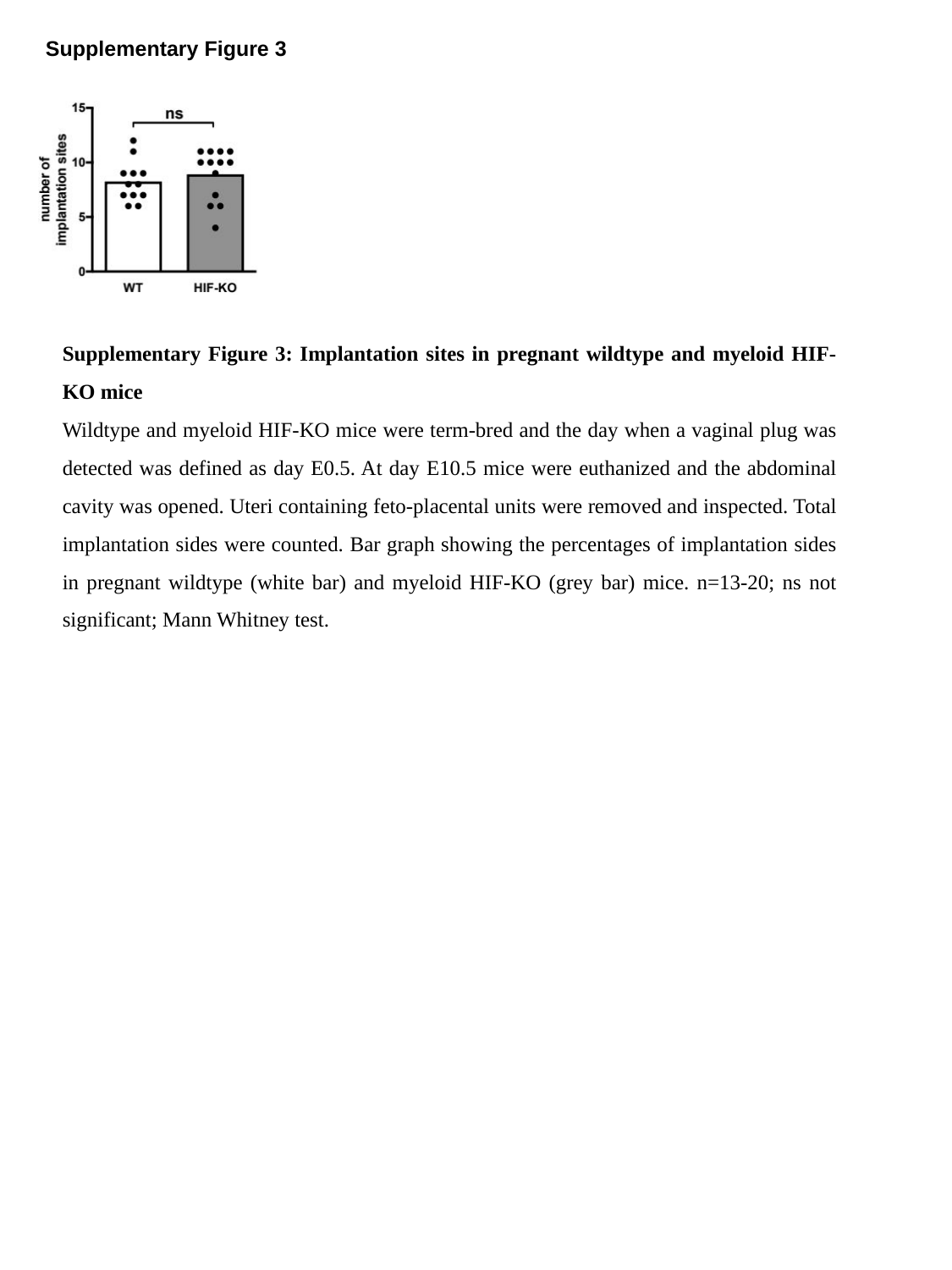

Supplementary Figure 3
Supplementary Figure 3: Implantation sites in pregnant wildtype and myeloid HIF-KO mice
Wildtype and myeloid HIF-KO mice were term-bred and the day when a vaginal plug was detected was defined as day E0.5. At day E10.5 mice were euthanized and the abdominal cavity was opened. Uteri containing feto-placental units were removed and inspected. Total implantation sides were counted. Bar graph showing the percentages of implantation sides in pregnant wildtype (white bar) and myeloid HIF-KO (grey bar) mice. n=13-20; ns not significant; Mann Whitney test.
